# A Rare Presentation of a Branchial Cleft Cyst: Can It Cause Syncope in a Pediatric Patient?

**DOI:** 10.7759/cureus.50004

**Published:** 2023-12-05

**Authors:** Fatemeh Akbarpoor, Asma Alshehhi, Khadeeja Aakef, Aftab Ahmed

**Affiliations:** 1 Medical School, College of Medicine, Mohammed Bin Rashid University Of Medicine and Health Sciences, Dubai, ARE; 2 Pediatric Surgery, Mediclinic Welcare Hospital, Dubai, ARE

**Keywords:** vasovagal syncope (vvs), congenital neck mass, carotid sinus syndrome, branchial cyst, branchial cleft cyst

## Abstract

Branchial cleft cysts (BCCs) are a congenital malformation most commonly seen in children and adolescents. BCCs are usually incidental findings and are benign in nature. In this report, we present a case of a 13-year-old female with syncope as a rare complication of a fluid-filled second BCC. The patient initially presented with a unilateral non-tender swelling on the right side of the neck and submandibular region, which was suspicious of an inflammatory process. After initial lab investigations came back negative, imaging studies of the neck with computed tomography (CT) with intravenous contrast revealed a hypodense lesion with a uniform density, which lay beneath the sternocleidomastoid muscle and abutted the carotid sheath. The cyst was surgically excised, and histopathological studies of the cyst wall and the analysis of the fluid contained within the cyst confirmed that it was indeed a branchial cleft cyst. We propose that the syncopal episodes she experienced most likely occurred due to the proximity of the cyst wall to the carotid sheath, which caused a mass effect leading to carotid sinus syndrome (CSS). This is the first case of CSS due to a BCC to be reported in the pediatric population.

## Introduction

Neck masses in children can usually be attributed to either inflammatory, neoplastic, or developmental causes. Most neck masses are benign and occur due to reactive processes in the lymph nodes, resulting in lymphadenitis. After thyroglossal duct cysts, branchial cleft cysts (BCCs) are the second-most common cause of a congenital neck mass, representing around 20% of pediatric cervical masses. Branchial cleft cysts are a rare developmental abnormality that is commonly seen in children but can also be seen in adolescents and adults. Branchial cleft sinuses and branchial cleft fistulas can also form as a result of incomplete obliteration of the cleft and pouches, with the latter being more common in younger children and infants. BCCs are often asymptomatic and benign in nature; they usually have defined borders and are painless and mobile. In the case of a superimposed infection, branchial cleft cysts may collect pus, form an abscess, and subsequently become tender [1].

BCCs are categorized into four different types based on their location; most cysts are second branchial cleft cysts, making up 95% of all BCCs, and are located beneath the sternocleidomastoid (SCM) and lateral to the carotid space [1]. First branchial cleft cysts are by far the rarest of all types and are mainly located superficially or deep within the parotid gland along the external auditory meatus. Third branchial cleft cysts are rarely encountered but are the most common posterior cervical congenital anomaly after thyroglossal ducts. These are situated more commonly on the left side and can extend starting from the posterior aspect of the carotid artery and can even pierce the thyrohyoid membrane. Finally, the fourth type is relatively rare and is encountered more on the left side. These are typically located in the thyroid gland or mediastinum and are discovered early in childhood [2,3].

BCCs can become infected, leading to the development of an abscess; hence, the recommendation is to excise the cyst entirely. Once a BCC is excised, recurrences are uncommon, but if there is previous surgery or recurrent infection within the BCC, the risk of recurrence can increase by up to 20% [3]. Notably, if BCCs get large enough, they can have a mass effect and press on surrounding structures in the neck, such as the carotid sheath and its contents, potentially leading to carotid sinus syndrome. To our knowledge, this complication was only discussed in two prior case reports to date [4,5].

## Case presentation

We report a case of a 13-year-old female who presented to the pediatric outpatient clinic with a non-tender right-sided mass in the upper third of the neck in addition to a mild sore throat. Her mother noticed a swelling developing approximately a month prior to presentation but was getting concerned since the swelling was enlarging. The patient denied fever, cough, fatigue, weight loss, palpitations, or breathlessness. Past medical history was significant for two syncopal episodes in the past year, as well as idiopathic adolescent scoliosis, which was diagnosed around a year ago. She reported undergoing regular physiotherapy sessions for her scoliosis, which she tolerated very well. She was eight months post-menarche and had no allergies. The patient had recently traveled to Sri Lanka; she also was quite athletic with swimming sessions at least twice weekly. 

Upon further questioning, her mother reported that the patient had experienced two unprovoked syncopal episodes in the last year, which were seven months apart; this was initially diagnosed as vasovagal syncope. The first episode was during a physiotherapy session for her scoliosis, where she felt dizzy and lost consciousness for 10 seconds, was sweating profusely, had a blood pressure of 87/48, and was tired when she regained consciousness. The second episode happened in the morning when she wanted to eat breakfast, was unsteady whilst walking, and lost consciousness for two minutes. She had never experienced these syncopal episodes while exercising or during periods of stress or anxiety. 

The patient’s vitals were within normal range. Further examination of the swelling showed that it was unilateral on the right side, in the submandibular and upper cervical regions, measured about 4 cm in diameter, had no overlying skin changes, was not tender, and had well-defined borders but slightly vague margins. The swelling was soft, fluctuant, and not transluminant. The pharynx was mildly erythematous, and the chest was clear to auscultation. Abdominal examination revealed that the abdomen was soft with no tenderness or distension, and there was no hepatosplenomegaly or groin swelling. Additionally, there were no palpable lymph nodes in the axillae bilaterally. 

Based on the patient's initial presentation, there was a high suspicion of benign lymphadenopathy or lymphadenopathy related to infectious mononucleosis due to the recent travel history and less likely a malignancy. Hence, cytomegalovirus (CMV) IgM antibody and three different Epstein-Barr virus (EBV) antibodies were ordered, namely anti-Epstein-Barr virus capsid antigen IgM (anti-VCA IgM), anti-early antigen IgG (anti-EA IgG) and Epstein-Barr virus nuclear antigen 1 IgG (anti-EBNA-1 IgG) to investigate the possibility of infectious mononucleosis as a cause. Additionally, full blood count (FBC), C-reactive protein (CRP), erythrocyte sedimentation rate (ESR), and peripheral smear of the blood were ordered. The blood test results are shown in Table 1. None of the tests showed evidence of an infectious or malignant etiology, as all the tests came back negative. This prompted an imaging study to be done to identify the nature of the lesion, starting with an ultrasound (U/S) of the soft tissues of the head and neck.

**Table 1:** Results of the initial investigations performed on the blood samples. Abbreviations: Cytomegalovirus (CMV); anti-Epstein-Barr virus capsid antigen IgM (anti-VCA IgM), anti-early antigen IgG (anti-EA IgG), Epstein-Barr virus nuclear antigen 1 IgG (anti-EBNA-1 IgG)

Test	Results
CMV IgM antibody	Non-reactive.
EBV antibodies	anti-VCA IgM, anti-EA IgG, and anti-EBNA-1 IgG were all nonreactive.
Full blood count	All values were within the normal range.
C-reactive protein (mg/L)	0.1
Erythrocyte sedimentation rate (mm/hr)	3.0
Peripheral smear	Red cells appeared to be normochromic and normocytic, with no abnormal forms seen. White cells were normal in morphology and maturation. Rare giant platelets were present. No blood parasite was seen.

**Figure 1:**
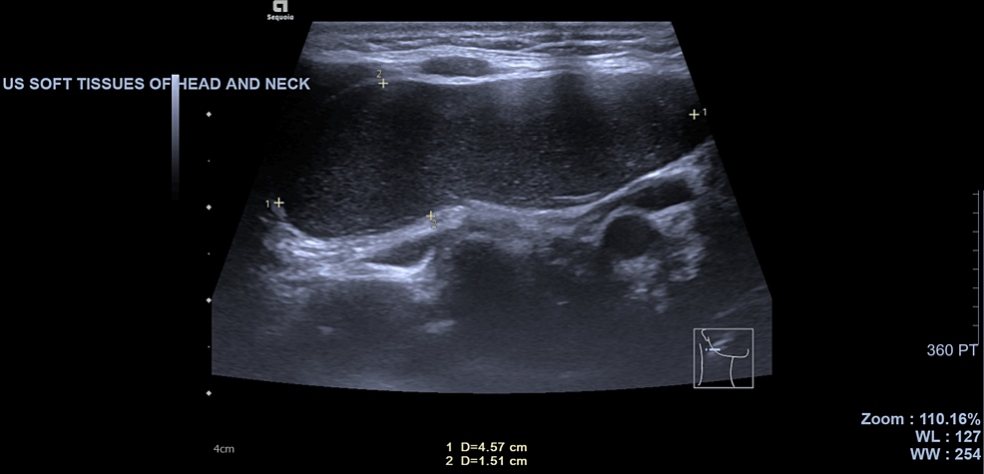
Ultrasonography of the right side of the neck and submandibular region showing the lesion's borders (marked with crosses).

**Figure 2:**
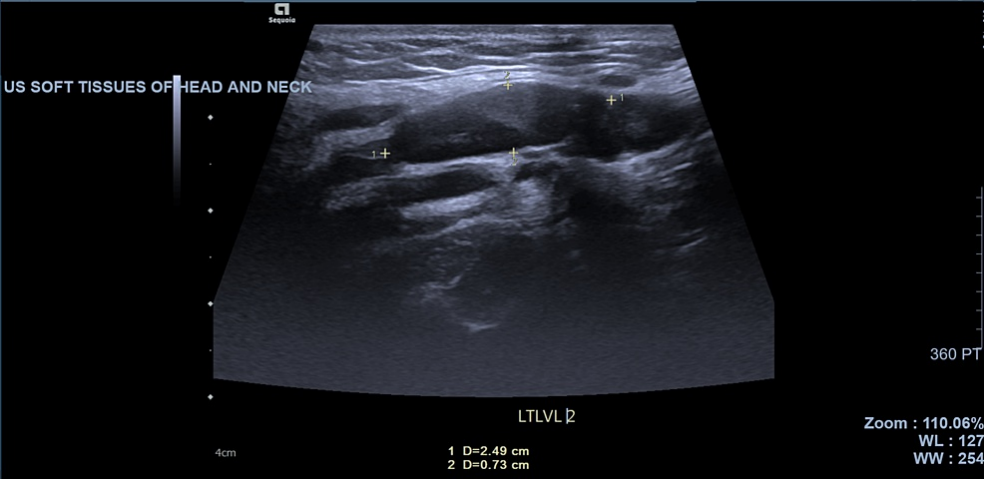
Ultrasonography of the left side of the neck showing left-sided level II lymph node prominence (borders marked with crosses).

Ultrasound imaging of the right side of the neck showed a large hypoechoic oval lesion in the right infra-auricular region, which could represent a branchial cleft cyst or enlarged lymph node (Figure 1). The parotid gland and submandibular glands on either side appeared normal. There was left-sided level II lymph node prominence, as shown in Figure 2.

**Figure 3:**
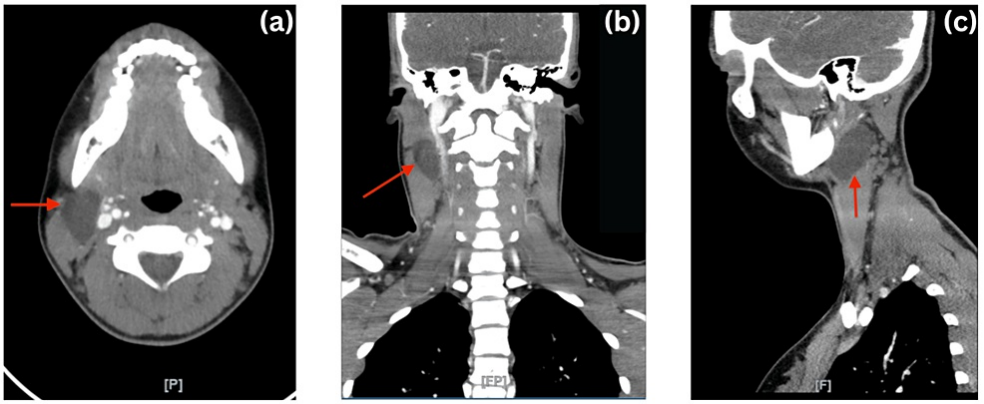
CT with contrast showing the lesion (red arrow) in the axial, coronal, and sagittal sections. The red arrow points towards the hypodense lesion, which measures 31x22x20 mm. The lesion had a uniform density and was observed in the right upper third of the neck, deep in the SCM muscle at the lower end of the parotid gland. It abutted the carotid sheath without infiltration and had neither calcifications nor septations.

After the ultrasound was performed, there was a recommendation from the radiologist to perform a CT scan of the neck and cervical spine with intravenous (IV) contrast to investigate the lesion further. The region of interest in the right upper third neck showed a fairly well-defined hypodense lesion with a uniform density, which was insinuated deep in the SCM muscle and the lower end of the parotid gland. This measured around 31x22x20 mm in size. It abutted the carotid sheath without any infiltration. No calcifications were seen in or surrounding the lesion. Figure 3 shows the lesion in the axial, coronal, and sagittal sections. There were multiple lymph nodes in the left posterior triangle of the neck as well as the jugulodigastric trunk, which were reactive in nature. 

The findings in this CT scan confirmed the presence of a cystic non-enhancing soft tissue mass in the right upper neck, which was highly suggestive of a branchial cleft cyst, most likely of the second type, and less likely necrotic lymph nodes. There was a surrounding mass effect exerted by the lesion but with no infiltration. The findings from the CT scan urged a referral to the pediatric surgeon.

Additional investigations were ordered to look for potential causes of the patient's recent syncopal episodes. Holter monitoring and electroencephalography (EEG) were ordered to look for cardiac or neurologic causes, respectively. Both of these came back normal.

The patient was referred to the pediatric surgeon, who recommended an excisional biopsy of the BCC under general anesthesia. An incision was made over the midpoint of the cyst, on the right side of the neck, to obtain access to the branchial cleft cyst. The epithelial wall was fully removed with caution to avoid injuring the surrounding structures. During the surgery, it was noted that the wall of the cyst was very friable and thin, and the cyst was filled with turbid fluid, which was aspirated to test for the presence of cholesterol crystals. This came back positive, which is pathognomonic of a branchial cleft cyst. Histopathological report of the cyst wall revealed that it was lined by stratified squamous epithelium, ciliated in some places, and surrounded by dense lymphoid tissue. This confirmed that it was indeed a BCC. 

The patient had a drain placed during the surgery to remove any residual fluid in the cavity, which was removed after one day; she tolerated the pain with the use of pain medication. She was also given one dose of an intravenous antibiotic as post-surgical prophylaxis. The patient had follow-up appointments at four and eight weeks after the operation. During both follow-up appointments, the incision site was noted to be healing well with minimal scarring, and the patient did not report any additional syncopal episodes when she was last seen by the surgeon. 

## Discussion

The primary differential diagnoses in a patient with a neck mass are congenital abnormalities, inflammatory causes, or neoplastic causes. The main etiologies to rule out in the case of a painless neck swelling are malignancies such as lymphoma or leukemia [1]. Additionally, it is important to rule out deep neck infections, such as a peritonsillar abscess, particularly in children presenting with fever. Malignancies usually arise with constitutional symptoms like fever, weight loss, or fatigue. While both lymphomas and benign cysts present with painless swellings, lymphomas are solid masses, unlike cysts which are usually soft. Certain features on ultrasound, such as lack of Doppler flow, can also aid in differentiating solid masses from cysts [6].

A BCC is a developmental abnormality resulting from the failure of involution of a part of the branchial apparatus; in this case, our patient most probably presented with a BCC of the second type [1]. The cyst lay adjacent to the carotid sheath without any infiltration. We propose that in our patient, the cyst was compressing the carotid sheath and causing carotid sinus syndrome (CSS), also known as carotid sinus hypersensitivity. This can be especially triggered by neck twisting and turning, which would increase the pressure from the BCC on the carotid sinus and potentially cause CSS [5].

**Figure 4:**
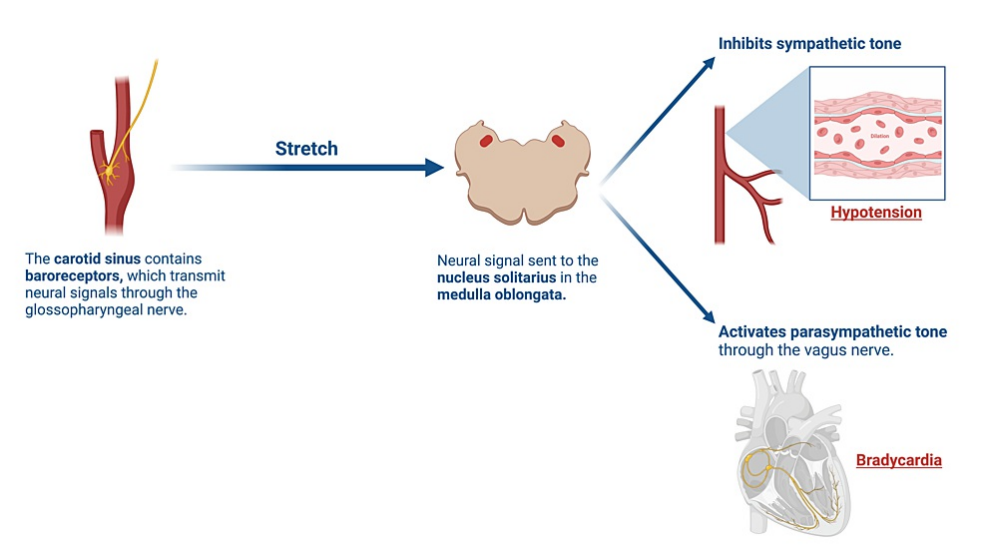
A simple illustration showing the neural pathway, starting from the baroreceptors in the carotid sinus and ultimately resulting in hypotension, bradycardia, or both. Created with BioRender.com

Carotid sinus hypersensitivity (CSH) is a condition that commonly manifests as syncope due to hypersensitive baroreceptors located in the carotid sinus at the bifurcation of the common carotid artery. When sensing a stretch, the baroreceptors send a neural signal through the glossopharyngeal nerve to the solitary nucleus in the medulla, which activates the parasympathetic output from the vagus nerve and inhibits sympathetic tone at the same time. This results in bradycardia due to parasympathetic output, hypotension, and vasodilation due to reduced sympathetic tone [7]. Hence, the proposed mechanism of CSH is due to excess stretching of or mechanical pressure on the carotid sinus, which overshoots signals, resulting in hypotension, bradycardia, or both [8]. These pathways are illustrated in Figure 4.

In light of the mechanism explained above, our patient's BCC was likely pressing on the carotid sinus, leading to increased parasympathetic tone and resulting in hypotension, bradycardia, and eventually syncope [8,9]. 

Whilst the syncopal episodes were initially thought to be a vasovagal reflex, this was ruled out as vasovagal syncope is usually preceded by a prodrome and is triggered by prolonged standing, emotional stress, or pain, which was not the case for our patient [10]. Both episodes were unprovoked and occurred in settings where the patient was not under imminent stress. It is also important to note that the patient is quite athletic and she did not get these episodes during any training sessions or periods of physical activity.

Carotid sinus syndrome is an infrequent complication of BCCs and was only seen in two previous adult patients with BCCs [4,5]. To our knowledge, this is the first pediatric case to be reported wherein a BCC was causing a syncopal episode attributed to CSH. 

## Conclusions

All in all, BCCs are benign masses seen in children and adolescents and are most commonly of the second type. When a pediatric patient presents with a neck mass, including BCCs in the differential list and considering possible complications is important. Excising the cyst fully is the mainstay treatment of a branchial cleft cyst to avoid superimposed infections, which can cause cyst enlargement, pain, and compression of surrounding structures. Although a rare complication, syncope and potentially life-threatening CSS can occur in patients with neck masses compressing the carotid sheath, such as a BCC, and should be taken into consideration when assessing a patient with an enlarging neck mass.
